# A brief review of complex regional pain syndrome and current management

**DOI:** 10.1080/07853890.2024.2334398

**Published:** 2024-04-03

**Authors:** Alaa Abd-Elsayed, Cain W. Stark, Natasha Topoluk, Mir Isaamullah, Paul Uzodinma, Omar Viswanath, Michael J. Gyorfi, Osama Fattouh, Kevin C. Schlidt, Omar Dyara

**Affiliations:** a Department of Anesthesiology, University of WI School of Medicine and Public Health, Madison, WI, USA; b Department of Anesthesiology, Medical College of Wisconsin, Wauwatosa, WI, USA; c Anesthesiology, LSU Health Sciences Center School of Medicine, New Orleans, LA, USA; d Department of Neurobiology, University of Wisconsin-Madison, Madison, WI, USA; e Department of Surgery, Sinai Hospital of Baltimore, Baltimore, MD, USA

**Keywords:** Complex regional pain syndrome, Budapest criteria, gabapentin, ketamine, Botulinum toxin A, antioxidants, sympathetic blocks, spinal cord stimulation, dorsal root ganglion stimulation, amputation

## Abstract

Complex regional pain syndrome (CRPS) is a debilitating chronic pain condition that, although exceedingly rare, carries a significant burden for the affected patient population. The complex and ambiguous pathophysiology of this condition further complicates clinical management and therapeutic interventions. Furthermore, being a diagnosis of exclusion requires a diligent workup to ensure an accurate diagnosis and subsequent targeted management. The development of the Budapest diagnostic criteria helped to consolidate existing definitions of CRPS but extensive work remains in identifying the underlying pathways. Currently, two distinct types are identified by the presence (CRPS type 1) or absence (CRPS type 2) of neuronal injury. Current management directed at this disease is broad and growing, ranging from non-invasive modalities such as physical and psychological therapy to more invasive techniques such as dorsal root ganglion stimulation and potentially amputation. Ideal therapeutic interventions are multimodal in nature to address the likely multifactorial pathological development of CRPS. Regardless, a significant need remains for continued studies to elucidate the pathways involved in developing CRPS as well as more robust clinical trials for various treatment modalities.

## SUMMARY

Complex regional pain syndrome (CRPS) is a debilitating and complex condition that places a significant physical, psychological and emotional burden upon afflicted patients necessitating multi-modal approaches to treatment.

The development of the Budapest criteria provided a robust and well-tested set of diagnostic criteria to aid clinicians in the diagnosis of CRPS.

The pathophysiology of CRPS has been challenging to elucidate with numerous proposed mechanisms, altogether suggesting a multi-factorial process is involved in the development of this condition.

Non-invasive treatments for CRPS are essential in addressing the physical limitations this disease can cause as well as addressing the significant psychological burden that involves increased incidence of depression and suicidal ideation.

Invasive treatments offer promising results, especially when considering dorsal root ganglion stimulation; however, the need for more robust clinical trials remains, especially when considering a small portion of patients who have refractory CRPS resort to amputation to control their pain symptoms.

## Introduction

Complex regional pain syndrome (CRPS) is a debilitating chronic pain condition that has been known by several names, including reflex sympathetic dystrophy or causalgia. First described in the sixteenth century, it was not recently that the Budapest criteria for diagnosis were developed to better enhance the specificity of diagnosing. Consisting of two general types delineated by the presence (CRPS type 1) or absence (CRPS type 2) of neuronal injury, further delineation of CRPS has been proposed although debate remains regarding these additional descriptors. An exceedingly rare condition affecting 6.28–26.2 per 100,000 person-years, CRPS is characterized by continuing pain, allodynia or hyperalgesia that is disproportionate to the inciting event with evidence of edema, skin or blood flow changes or abnormal sudomotor activity in the region of pain with a predominance for the distal extremities [1]. Despite the development of the Budapest criteria, the challenge with diagnosing CRPS remains due to a lack of understanding regarding the underlying pathophysiology. Multiple etiologies have been proposed and its mechanism of onset is likely multifactorial in nature given the complexity of symptoms. Most often, patients will develop CRPS after fractures or blunt traumatic injuries. In a retrospective review, a rare subset of patients developed CRPS spontaneously without any memorable underlying event [2]. The predominance for preceding fracture or blunt traumatic injuries suggests a higher prevalence in athletes, a consideration that should be given to that particular population since earlier diagnosis allows for a greater likelihood of response to treatment modalities [3]. Given the currently elusive nature of CRPS pathophysiology, it behooves a multimodal approach to treatment with modalities therefore remaining broad, ranging from a variety of non-invasive and invasive modalities [4–6].

## Classifications

CRPS has previously been known by other names, usually reflex sympathetic dystrophy or causalgia. It first entered the medical lexicon in 1994 at a meeting in Orlando where the International Association for the Study of Pain convened and defined CRPS. This led to the categorization of Type 1 and Type 2 CRPS being separated by the presence of a defined nerve lesion (Type 2) [7]. A number of society consensus meetings, most recently in 2007, have resulted in a more standardized common methodology for the diagnosis of CRPS [8]. This resulted in what is now known as the Budapest Criteria, a more widely accepted set of diagnostic criteria for CRPS. This requires continuing pain disproportionate to any inciting event; one symptom in three of the following four categories: Sensory, Vasomotor, Sudomotor/Edema, Motor/Trophic; one sign at the time of evaluation from the same categories; and there must be no other diagnosis which better explains these signs and symptoms. At the same Budapest conference, the decision was made to keep the distinction of CRPS Type 1 and Type 2, again delineated by the absence or presence (respectively) of known nerve lesion, though of possibly limited clinical utility.

Looking at the role of the autonomic nervous system in CRPS however may help us make a more useful clinical distinction however. When tissue injury occurs, regardless of whether direct peripheral nerve injury occurs as well, there is an upregulation of proinflammatory factors and sympathetic nervous system noradrenaline receptors [9]. Additionally, in the milieu of recently damaged tissue, there is evidence of dysfunction in the autoinflammatory and autoimmune responses [10]. This leads to an exaggerated response to endogenous catecholamines in the post-injury period, again regardless of direct peripheral nerve injury. This may explain the periods of sweating, warmth, swelling and inflammation, alternating with periods of coolness (as the catecholamine/autonomic response waxes and wanes) that is not consistent with the normal expected course of tissue healing after trauma. As the post-trauma period continues, patients with CRPS tend to develop into either a Warm or Cold type of CRPS. This classification of Warm versus Cold CRPS was recently explored in a larger multisite study which sought to better define these subtypes and their prognostic implications. They found that there is a Warm subtype that is predominantly patients with acute CRPS which resolves within 6 months, while the Cold subtype is associated with a chronic type of CRPS [11]. These classifications of Warm or Cold CRPS thus are independent of classification as Type 1 or Type 2 CRPS as they are not explicitly related to the evidence of direct peripheral nerve injury. Furthermore, it must be emphasized that these stages are not necessarily progressive, but related to time intervals from initial insult or onset [12].

## Pathophysiology

As previously noted, CRPS can be classified into two main categories: type 1 and type 2. Type 1 is also known as sympathetic dystrophy and is notable for there being no neural injury involved in the pathogenesis. In contrast, type 2 is a result of nerve damage [13]. Pathogenesis is still unknown, yet there have been several studies conducted which have revealed some important mechanisms that are involved with it.

Type 1 CRPS has two agreed upon clinical stages, with the first stage being the acute ‘warm’ phase and the second stage referred to as the chronic phase, also known as the ‘cold’ phase. In the acute ‘warm’ phase patients show clinical signs of inflammation, edema, pain and change in skin temperature in the affected limb [13,14]. Studies show that the inflammation could be caused by an amplification of the innate immune response that leads to the proliferation of skin cells such as keratinocytes that are responsible for releasing pro-inflammatory cytokines TNF-alpha, IL-1B and IL-6, thus triggering an inflammatory response [6,15]. The release of cytokines results in the symptoms seen in the effected limb during the warm phase of CRPS such as vasodilation, swelling, warmth, and pain [16]. More evidence to this comes from a meta-analysis of studies that investigated the classic mediators of inflammation in CRPS. These studies found that IL-8 levels, a biomarker for the inflammatory response, were elevated significantly during the acute ‘warm’ phase of CRPS [13,17,18]. In multiple studies it was found that CD4+ and CD8+ lymphocyte population were increased suggesting a response from antigen-mediated T lymphocytes [6,19,20]. Analysis of serum levels of pro-inflammatory and anti-inflammatory cytokines showed IL-37 as a biomarker for the immune response. It was found that there was decreased serum levels of IL-37 as well as an increase of GM-CSF showing pro and anti-inflammatory cytokine response in CRPS with pro-inflammatory dominating the pathogenesis [16,20]. Several studies posit that exaggerated inflammatory response in the setting of impaired healing can help define or establish a CRPS course. Inflammatory cytokines may also be implicated as there is some evidence from elevated levels of IL-1B and IL-6 in cerebrospinal fluids from chronic CRPS patients while other studies by do not agree [21–23]. Interestingly, several studies have noted the observed inflammatory-mediated changes in both peripheral and central sensitization are not due to cell-mediated immune responses as they have reported normal levels of cell markers such as lymphocyte counts, sedimentation rates, antibody levels, activated T-cells and blood cell counts [24–26]. The exact role of cell upregulation or activation therefore remains unclear.

The chronic phase is associated with a reduction in the inflammatory component but persistence of pain [13]. Despite the decrease in inflammation seen in the chronic ‘cold’ phase of CRPS, pro-inflammatory mediators such as IL-6, MCP-1, and MIP-1B remain elevated [13,27]. In fact, some of the changes and symptoms seen in the chronic phase are the result of elevated inflammatory markers noted in the acute phase. The pro-inflammatory cytokines in the acute phase, including TNF-alpha, IL1B, and IL-17, activate osteoblasts and osteoclasts which result in osteoporotic changes due to rapid bone turnover [14,28–30].

Neurogenic inflammation plays a key role in the development of CRPS symptoms such as allodynia and hyperalgesia. The peripheral nociceptive C-fibers are stimulated, resulting in the conduction of the stimulus both afferently to the dorsal ganglia as well as efferently to the affected tissue. This retrograde transmission causes the release of pro-inflammatory neuropeptides substance P, calcitonin gene-related peptic (CGRP), as well as adrenomedullin, neurokinin B, vasoactive intestinal peptide, neuropeptide Y and gastrin-releasing peptide [13,31]. Neuropeptide substance P and CGRP would bind to their respective receptors on keratinocytes leading to proliferation and neurogenic inflammation [6,32]. Substance P and CGRP also activate mast and dendritic cells which release histamine, serotonin and TNF alpha resulting in the attraction of inflammatory cells to the area in which they were released, resulting in further inflammation [33].

Multiple studies have shown evidence of an autoimmune component to CRPS. It was found that 35-40% of CRPS patients show surface binding autoantibodies targeting their sympathetic neurons, mesenteric plexus neurons, and cholinergic type cell lines [34,35]. Additionally, collected samples of serum, skin, and tissue from CRPS patients were found to have an elevated level of autoantibodies [6,36]. In an experiment conducted using mice injected with the IgG serum from a CRPS patient, findings were notable for those mice subsequently experiencing hypersensitivity to painful mechanical stimuli but not to tactile stimulation [37]. Additionally, the increased IgM levels that were found in the skin of CRPS patients as well as in the spinal tissue of rats, is thought to be responsible for the increased nociceptive sensitization.

Specifically, the elevated deposits of IgM in the skin could be caused by the release of neuropeptides during the retrograde transmission seen in the C-fibers during neurogenic inflammation [33]. Further evidence to support the autoimmune component involves dendritic cells which are stimulated by IgG complexes resulting in the migration of the dendritic cells to draining lymph nodes from peripheral tissue [14,38]. Furthermore, there may be genetic dispositions as noted by polymorphisms in tumor necrosis factor-alpha (TNF-alpha) and human leukocyte antigen (HLA) which may contribute to an earlier age of onset [39].

Increased Langerhans cell numbers were also found in affected skin samples of patients with CRPS, yet patients with chronic CRPS showed fewer Langerhans cells [40]. However, studies performed comparing wildtype and Langerhans cell knockout animals sensitization found no difference between the two groups when testing sensitization [33]. Notably, Russo et al. argues that the significance of the difference in the numbers of Langerhans cells found in the Osbourne study is important. Citing Langerhans cells migrating to the dermis and draining the lymph node results in the priming of T-Cells to initiate an immune response [41,42]. Regardless of the role of Langerhans cells, there is a clear need for additional research to better understand the role Langerhans cells potentially play in the immunological response in CRPS pathogenesis.

### Sensitization in CRPS

As previously noted, the typical classification of CRPS into type 1 and 2 can be ambiguous. The former is associated with noxious stimulus while the later with aberrant nerve. In CRPS, the constant noxious stimuli can result in peripheral and central sensitization. The sensitization is demonstrated by an enhanced level of sensation either from heightened pain (hyperalgesia) and unprovoked pain (allodynia). The states of hyperalgesia and allodynia is further explained by the aberrant interpretation by somatosensory units of the central nervous system (CNS) of benign stimuli such as light touch as pain. The skin over, and even beyond the initial area of insult, can become affected by the aberrant pain perception reflecting the characteristic regional presentation of this chronic pain syndrome in addition to other changes that reflect sensory, peripheral and sympathetic nervous system dysregulation [43].

The pathophysiology of peripheral and central sensitization underscores changes in the sympathetic nervous system (SNS) with resultant localized heightened sympathetic activity. Activated peripheral nociceptors that convey pain from either chemical, mechanical or thermal stimuli *via* unmyelinated C fibers and partially myelinated A-delta fibers that project to the Rexed layers I, II, and V in the spinal cord activate the release of excitatory amino acids glutamine and asparagine. These amino acids serve as ligands on *N* –methyl- *D* –aspartic acid (NMDA) receptors, further resulting in the release of substance P [44].

### Peripheral sensitization

In peripheral sensitization, repetitive noxious stimulation of C fibers results in heightened sensitivity, coupled with reduced stimulus thresholds and prolonged activation of dorsal horn cells, particularly those with excitatory glutamine receptors. The resultant excitation is largely due to a myriad of inflammatory mediated processes that includes actions by substance P (SP), calcitonin gene-related peptide (CRGP), leukotrienes, prostaglandins, histamine, bradykinin, and serotonin, all of which have versatile ligand properties that further compound the inflammatory process and algogenic states [45].

It is known that tissue trauma can cause the release of cytokines and nerve growth factor (NGF) which further activate nociceptors by stimulating the release of inflammatory or excitatory amino acids and neuropeptides in primary afferent neurons as previously noted thus contributing to *long-term* peripheral sensitization [46].

### Central sensitization

In the pathological spectrum of CRPS, several mechanisms underlying central sensitization have been posited to include not only the state of enhanced excitability of neurons in the spinal cord but also inclusive of neuroplastic alterations in the somatosensory cortex and endogenous pain modulations [45].

The role of the central nervous system (CNS) cannot be overemphasized as it is thought to undergo structural and functional changes in CRPS patients. In particular, there are plastic changes such as the excitability of thalamocortical nociceptive pathways due to loss of peripheral inhibition and upregulation of glutamate receptors domicile in the CNS [47,48]. In addition, other neuronal plastic changes confer motor dysfunction in CRPS patients as evidenced by increased dystonia in the population [49]. The exact mechanism underlying dystonia in these patients is still unclear as several studies have only suggested an association.

Furthermore, an inherent characteristic of central sensitization is *mechanical hyperalgesia.* It has been posited that this phenomenon is due to the activity of second-order nociceptor specific neurons and particularly wide-dynamic-range (WDR) neurons in the spinal cord contribute to an expansion of pain sensation beyond the initial loci of injury. There is a compounding of effects due to contributions from peripheral inputs (sensitization) that creates a crescendo-like amplification of pain patterns [50].

One of many classic representations of peripheral contribution to CNS amplification is seen with excitatory neurons mediated-disinhibition of both trigeminal and spinal mechanical neurons and or excitatory neurons mediated-facilitation of nociceptive activity which project from the rostroventral medulla [51].

### Autonomic

The old dogma on the etiology of CRPS was the role of sympathetic nervous system (SNS) hyperactivity and the unique pattern of pain observed in patients was thought to be sympathetically mediated. While current literature highlights numerous addenda to this founding posit, it nevertheless reflects a more complex etiology that has been recently explained as an unusual pathological synergy between nociceptive afferent neurons and sympathetic efferent neurons.

Some physiologic changes have been observed particularly in the acute and subacute phase of CRPS, including aberrant skin color, temperature, sweating from vasodilatation, edema, hypertrichosis, hyperhidrosis, hypohidrosis, accelerated or decelerated nail [49]. Furthermore, the vasodilatory signs observed in acute settings suggests an inhibition/denervation of sympathetic action in the short-term that eventually results in hypersensitivity and upregulation of adrenergic receptors such that in subacute and chronic phase of CPRS, the presence of circulating catecholamine results in vasoconstriction as manifested as coolness. The psychological distress of the pervasive pain and autonomic symptoms further enhances catecholamine release and potentially exacerbates the aforementioned pathophysiologic mechanisms. In one study, a significant positive correlation between Beck Depression Inventory scores and catecholamine concentration was identified, further supporting the potential augmentation of CRPS pathways and subsequent symptoms [52,53].

The unusual synchrony or coupling between the nociceptive afferent and sympathetic efferent neurons have been described as likely to be a norepinephrine-mediated interaction between the afferent somata within the dorsal root ganglion (DRG) and sympathetic vasoconstrictor neurons, or between regenerating or intact peripheral nociceptive C-fiber and sympathetic efferent neurons [54]. mRNA activity for alpha −2-adrenergic receptors have been described in DRG neurons of injured nerves as contributing to vasodilatation [55].

## Presentation and diagnosis

### Historic considerations

Presentations consistent with CRPS have been described as early as the sixteenth century. Amroise Paré is often credited with the first account of CRPS when he described persistent extremity pain experienced by King Charles IX after bloodletting [56]. In the early 1800s, Denmark reported a case of unrelenting burning pain after a gunshot wound necessitating upper extremity amputation [57]. During the civil war era, Mitchell and colleagues described multiple accounts of trauma-induced intractable burning pain [58]. Mitchell, aware of the afore mentioned, coined the term causalgia to describe such presentations. However, it was not until the early 1900s that the first diagnostic criterion was described. This criterion highlighted the spontaneous and burning characteristics of the pain while noting the tendency for painful exacerbations and mental status changes [59]. As early as 1916, there were speculations that the underlying mechanism of such presentations involved over excitation of the sympathetic nervous system [60]. Sudek and Evans described associated edema and motor limitations with or without the presence of an identified nerve lesion, naming such syndromes reflex sympathetic dystrophy (RSD) and Sudek dystrophy/atrophy, respectively [61,62].

It was not until 1979 that the International Association for the Study of Pain (IASP) formally defined these syndromes as sustained burning pain after a traumatic injury involving vasomotor, sudomotor and trophic changes with similar presentations but different causes [63]. In 1994 the IASP accepted and published the descriptor ‘complex regional pain syndrome’ to describe a condition clinically defined by: (1) The presence of an initiating noxious event, (2) continuing pain, allodynia or hyperalgesia, which is disproportionate to the inciting event, (3) evidence of edema, skin or blood flow changes or abnormal sudomotor activity in the region of pain, (4) the diagnosis is excluded by the existence of conditions that would otherwise account for the degree of pain and dysfunction [64]. These criteria, known as the Orlando criteria, were found to have high sensitivity but poor specificity on internal and external validation testing, leading to inadvertent over diagnosis of CRPS [59].

### The Budapest criteria

At the consensus meeting of the IASP in Budapest in 2004, new criteria for the diagnosis of CRPS were developed [8]. In an effort to enhance specificity, these criteria included both symptoms and signs of the disease. These criteria, known as the Budapest criteria, are listed in Table 1. Concordant with their objective, validation testing revealed a sensitivity of 0.76 (formerly 0.98 with the Orlando criteria) and a specificity of 0.81 (formerly 0.36 with the Orlando criteria) when utilizing 3 of 4 symptom and 2 of 4 sign decision rules for diagnosis [65]. The Budapest criteria were formally accepted by the IASP in 2012 after more robust validation testing [66]. It should be noted that a 4 of 4 symptom decision rule is recommended when applying the criteria for research purposes [65].

**Table 1.** Budapest criteria.

Procedure Standards*	I. All symptoms criteria should be asked at each formal evaluation II. For unilateral CRPS, assess asymmetry via comparison with the contralateral extremity III. For bilateral CRPS, assess for changes relative to an unaffected extremity or assess for asymmetry relative to a typical, healthy individual Iv. The full criteria must be applied to each individual extremity v. In the event an examiner observes a sign that the patient does not express as a symptom, a “signs over-rides symptoms” approach cannot automatically apply^#^	
The Budapest Criteria	For the clinical diagnosis of CRPS, all of the following must be true:	
	I. Patient describes continuing pain, disproportionate to the inciting event	
	II. Patient reports at least one symptom in all four categories 1. Sensory a. Allodynia b. Hyperalgesia 2. Vasomotor a. Temperature asymmetry b. Skin color changes c. Skin color asymmetry 3. Sudomotor a. Change in or asymmetrical edema b. Change in or asymmetrical sweating 4. Trophic a. Decreased range of motion b. Weakness, tremor, dystonia c. Changes of hair, nail, skin	
	III. Physician observes at least one sign in at least two categories 1. Sensory a. Allodynia to light touch (light manual touch or brush*), temperature, somatic pressure (application of pressure until the examiner’s nail bed turns white, roughly 100g/cm^2^*) or vibration (assessed with a graded tuning fork over a bony prominence*) b. Hyperalgesia to pinprick (applied centrally in most affected extremity and compared to the same location in the unaffected extremity*) 2. Vasomotor a. Temperature asymmetry of >1 °C (If quantitative metric is not obtainable, temperature change should be obvious to examiners when touching the area with the dorsum of their hands*) b. Skin color changes c. Skin color asymmetry 3. Sudomotor a. Change in or asymmetrical edema b. Change in or asymmetrical sweating 4. Trophic a. Decreased range of motion b. Weakness, tremor, dystonia c. Changes of hair, nail, skin	
	IV. No other diagnosis better explains the described symptoms and observed signs	
Corresponding Diagnosis	CRPS Type I	If the criteria are met at the time of evaluation and there is no known nerve lesion in the affected area
	CRPS Type II	If the criteria are met at the time of evaluation in an anatomic region extending beyond the territory of any identified nerve injury*
	CRPS with Remission of Some Features*	If the medical record confirms the criteria have been met at some historical point. However, the criteria are not met at the time of evaluation
	CRPS Not Otherwise Specified	If the medical record confirms the criteria have never been met.* However, there is no alternative diagnosis that better explains the presentation

Adapted from Harden et al.

^*^Indicates taxonomic or procedural clarification described by Goebel et al.

^#^See Goebel et al for examples of such situations and recommended action.

In 2019, the IASP CRPS special interest group convened for a discussion of perceived ambiguities in the Budapest criteria for CRPS diagnosis. This resulted in a consensus-based adaptation of diagnostic taxonomy, which represents a clarification of CRPS assessment instructions without changing the diagnostic criteria, itself [67]. Notable areas of clarification include: (1) recognition that CRPS 1 and 2 have identical diagnostic signs. For correct diagnosis of CRPS 2, the diagnostic signs must extend beyond the territory of any identified injured nerve (2) the diagnosis ‘CRPS Not Otherwise Specified’ should only be applied to patients who have never fulfilled the Budapest criteria for CRPS diagnosis but whose condition is not better explained by another disease process (3) definitions for asymmetry, hyperalgesia, and allodynia, as they relate specifically to CRPS diagnostic assessment. While this expert group acknowledged the importance of distinguishing warm and cold variations of CRPS, they did not feel there was sufficient data to support a consensus adoption of these variations to formal CRPS subtypes. They did, however, introduce a new CRPS subtype, CRPS with Remission of Some Features, which represents a patient population not currently meeting the Budapest criteria but who have a documented history of meeting the criteria in the past. The group acknowledged that future work would focus on further defining the diagnostic transition to this subtype.

The Budapest criteria remain the gold standard for the clinical diagnosis of CRPS. The criteria are endorsed by the IASP and all its affiliate federations and chapters. Multiple researchers and physicians have proposed other criteria for the diagnosis of CRPS, but none of these have undergone and withstood robust validation testing as successfully as the Budapest criteria [24,68–73].

### Utility of diagnostic testing

No singular objective test is specific for CRPS. Diagnostic standards for many pain organizations, including the European Pain Federation, support the use of diagnostic testing as a means to exclude differential diagnoses [74]. A majority of those ultimately diagnosed with CRPS have undergone extensive diagnostic testing including but not limited to the following: bone scintigraphy and other bone densitometry studies have been reported to show increased uptake in CRPS affected joints [75]. It is generally recognized that earlier use of tri-phase bone scintigraphy correlates with increased specificity of the study for CRPS. Compared to other utilized objective tools, these studies are relatively easy to obtain in clinical practice. Multiple meta-analyses have concluded scintigraphy should not be used to rule in disease; however, it could have utility as a confirmatory test [76,77].

Doppler flow studies have been suggested to assess vascular reflexes, especially in patients with symptom duration of four months or less [65]. It can also be used for exclusion of contributing vascular pathologies, particularly venous thrombosis.

Infrared thermometry/thermography has the highest specificity of any objective test for CRPS [65]. In earlier phases of the disease, the affected limb typically shows higher temperatures compared to the contralateral control [78]. This observation has been shown to reverse with disease progression. Sensitivity for CRPS has been reported as low as 45% [63]. Thermography requires specialized equipment and accuracy depends on the ability to maintain thermoregulation during measurements [65,79]. The utility of other autonomic function tests including, quantitative sudomotor axon reflex testing and thermoregulatory sweat testing, have similar limitations in that they require specialized equipment, knowledgeable operators, and they have a lower specificity for CRPS [63,65].

Magnetic resonance imaging (MRI) is especially useful in the exclusion of musculoskeletal disorders, particularly osteonecrosis [63]. MRI has been shown to be less sensitive for CRPS diagnosis than bone scintigraphy in comparative studies [80].

Plain radiography is one of the most affordable and easiest objective tests to obtain. Films often show a heterogeneous demineralization of bone in endorsed painful regions. However, x-ray has low reported sensitivity and specificity for CRPS [61,63]. Its greatest utility is arguably exclusion of other musculoskeletal injuries, particularly in early diagnosis of a unilateral extremity presentation of the disease.

### Limitations of current diagnostic standards

As previously discussed, CRPS remains a clinical diagnosis of exclusion. Accurate diagnosis relies on standardized assessment and application of diagnostic criteria across physicians, which has historically been limited. Further taxonomic clarifications and standardization of assessment techniques, like those described by the CRPS task force in Valencia, represent necessary strides forward towards more uniform and generalizable diagnostic criteria. Specifically, clarification of prior definitions such that CRPS type 2 must include diagnostic signs that extend beyond the identified injured nerve territory. Additionally, the development of a third category, CRPS Not Otherwise Specified (NOS) allows for the inclusion of patients who had previously met diagnostic criteria but do not meet the updated diagnostic criteria put forth by the Valencia task force. Furthermore, the updated diagnostic criteria require patients to report at least one symptom in three or more of the four categories whereas previously they were required to have symptoms in all four categories outlined in the Budapest criteria. The task force also highlights a need to clarify if CRPS type 1 and 2 are truly unique diagnoses, as well as the need for sub-group identification (warm vs cold, for example) is critical to management [67].

## Treatment

### Non-invasive interventions

#### Physical, occupational and psychological therapy

The role of physical and occupational therapy (PT and OT, respectively) is part of a multi-modal approach to the management of CRPS and is often a keystone in a patient’s treatment regimen [4]. The challenge with implementing PT and OT is the broad range of interventions available to therapists and the persistent lack of understanding regarding the underlying pathophysiology of CRPS. Regardless, a number of case reports have been published showing efficacy in a wide range of interventions, ranging from strain-counterstrain (SCS), neural mobilization techniques, and thrust manipulation, to name a few [81–83]. These techniques often rely on some form of neuromodulation, either through decrease in proprioceptive hyperactivity as seen in SCS or activation of descending pain pathways and inhibitory mechanisms in spinal manual therapy. Additionally, there is evidence of analgesic effects achieved through the release of neurotensin and oxytocin, providing patients with short-term analgesia that may subsequently enhance their ability to participate in other therapeutic modalities they may previously not have tolerated [84,85]. Additionally, as previously noted, there is likely a role involving catecholamines in the pathophysiological pathway of CRPS. Although the evidence is lacking in clinical trial evidence, case series have shown promise in the benefits of various forms of psychotherapy intervention, especially when combined with other non-invasive modalities. Additionally, adaptation of the treatment regimen to the evolution of a patient’s symptoms is critical [12]. Of particular interest is the study conducted by Fialka et al. who found a decrease in limb temperature in the psychotherapy arm compared to the physical therapy only group. Although the study was limited by its lack of power, it lends further support to the role psychological stress can play in the progression of CRPS [86,87]. Furthermore, this particular patient population carries significant risk factors for suicidal ideation, including: severe pain, depressive symptoms and functional impairment. The link between chronic pain and suicidal ideation has been well established, and given the additional autonomic dysfunction associated with CRPS, these patients carry a significant risk for suicidality [88–91]. The range of benefits is variable and there remains a paucity of evidence regarding these modalities; however, given the current understanding and proposed pathophysiological mechanisms, these interventions are logical in their employment.

### Medical management

#### Non-steroidal anti-inflammatory drugs

Non-steroidal anti-inflammatory drugs (NSAIDs) are cyclooxygenase (COX)-2 inhibitors, preventing the synthesis of prostaglandins. Prostaglandins are inflammatory mediators and induce hyperalgesia; therefore, downregulating these molecules may disrupt the process of spinal transmission of nociceptive signals and reduce the acute-pain stage of CRPS [92]. However, the evidence remains poor regarding the efficacy of this drug class although there are case reports showing potential benefits [6,93].

#### Steroids

Steroids are used to target the inflammatory component of the proposed CRPS pathophysiology mechanism. There have been several studies assessing the efficacy of steroids, at various doses, various time-intervals and in patients with varying degrees of CRPS duration (acute vs chronic) [94–96]. Given the noteworthy and potentially significant adverse effects associated with prolonged steroid use, care needs to be exercised by the clinician when considering these medications. One trial in particular was notable for the significant benefits of a tapered prednisone regimen in patients with an average duration of 1.9 months since onset of CRPS in reducing VAS pain scores [97].

#### Gabapentin

Gabapentin, originally used as a muscle relaxer and anti-spasmodic medication, has been found to be an effective adjunct anticonvulsant with neural pain control potential [98]. Van de Vusse et al. performed a randomized double blind placebo controlled crossover study and found that while gabapentin only had a mild effect on pain in CRPS I, it had a significant impact on reducing the sensory deficit in the affected limb [99]. These results were similar in the pediatric population, as Brown et al. found that gabapentin significantly reduced pain intensity scores while improving sleep [100]. This study found that both the antidepressant amitriptyline and the anticonvulsant gabapentin were analogous in their effectiveness in reducing pain in CRPS I. A limitation of these trials was the low number of participants in the trials. While commonly utilized in the treatment of CRPS, there are ultimately few randomized control trials with solid evidence to prove the efficacy of gabapentin, suggesting that there needs to be further research into gabapentin with respect to CRPS [4].

#### Bisphosphonates

Bisphosphonates inhibit osteoclastic bone resorption by to hydroxyapatite sites, especially at sites undergoing active bone resorption. The exact mechanism for how bisphosphonates provide analgesia in CRPS remains unclear, although there are a number of potential mechanisms that have been proposed and the true mechanism is likely multifactorial in nature given the limited degree of bone turnover noted in CRPS [101]. Regardless, there appears to be a degree of evidence indicating the benefit of bisphosphonates in reducing pain scores in comparison to placebo; however, many of the early trials were often lacking in power. In addition, a lack of true understanding behind the mechanism of action for therapeutic benefit with bisphosphonates limits the ability to identify ideal drug and dosing regimens [4,102]. However, a randomized, double-blind, placebo-controlled study comparing four doses of IV neridronate to placebo found statistically significant decreases in VAS scores in the study of 82 patients. The authors posit that these results may be linked to bisphosphonate reduction of the increased bone turnover and subsequent marrow edema seen in CRPS that may contribute to chronic pain. Additionally, locally accumulated drug may interfere with the inflammatory pathways and subsequent pain generation by decreasing lactate concentration and acidosis. Interestingly, some patients also experienced permanent remission of their disease; however, it should be noted that these patients were identified early in their clinical course, an important caveat to the efficacy of bisphosphonates in treating CRPS. Regardless, the significant efficacy and limited adverse effects of the medications suggest an importance in incorporation of bisphosphonates as standard of care when initiating treatment for CRPS patients [103].

#### Ketamine

Ketamine is an N-methyl-D-aspartate (NMDA) receptor antagonist with a notable role in the modulation of the wind-up response to noxious stimuli that results in central sensitization classically seen in chronic pain conditions. There is also evidence of a role in immunomodulation resulting in the down-regulation of neuroinflammatory markers; therefore, it remains unclear if the potential role of ketamine in CRPS treatment is relegated solely to the effects of NMDA antagonism resulting in down-regulation of central sensitization or if there are additional factors involved [104,105]. Regardless, the current body of evidence is lacking in supporting the efficacy of ketamine in the treatment of CRPS with the majority of studies lacking in power or utilizing different modes of administration, making it difficult to determine the level of evidence supporting ketamine [5,105,106].

#### Botulinum Toxin A

The use of Botulinum Toxins A and B have been an emerging adjunct to local anesthetics for sympathectomies and treatment of dystonia/spasticity in the treatment of CRPS, with most studies published starting in 2008. The mechanism of this therapy was hypothesized to be slightly different depending on the use for treating dystonia and spasticity or for a sympathectomy. In dystonia, the role of botulinum toxin in therapy is due to its function in disrupting the SNARE complex and inhibition of exocytosis. It prevents the release of acetylcholine and causes flaccid paralysis, effectively relieving the pain from muscle dystonia and spastic contractility [107,108]. A retrospective case review of the use of Botulinum Toxin A (BTA) in patients with neck or upper limb girdle muscles with dystonia and spasm showed that there was significant pain relief in 97% of the patients over the first 4 weeks after treatment. Given the efficacy of botulinum toxin in inhibition of cholinergic circuitry, several studies have expanded its use to blocking cholinergic signaling at the sympathetic ganglia in CRPS patients. The majority of studies have been small case-control or retrospective studies regarding the efficacy of Botulinum Toxin A (BTA) and Botulinum Toxin B (BTB), either by themselves or in conjunction with local anesthetic. While these studies range from retrospective studies to prospective randomized controlled trials, they all show moderate improvements in pain scores over the short term with either botulinum toxin alone or with local anesthetic. They also reliably show that the use of botulinum toxin has a greater duration of efficacy than the use of local anesthetic alone [109]. A meta-analysis performed in 2020 which evaluated randomized controlled trials provided good evidence for the efficacy of BTA for pain relief across neuropathic and muscle based pain syndromes [110]. There are numerous other studies that suggest the use of BTA or BTB depending on the location of the sympathectomy, but there is not yet strong evidence for the use of one or the other purely based on the site of CRPS being treated [111,112]. There is currently a study underway with very similar parameters looking at the impact to quality of life and disability scores in CRPS patients [113]. While BTA has significant therapeutic potential, there are concerns about possible extravasation into surrounding ganglia which could lead to neurologic complications as well as discrepancies between industry and non-industry-sponsored studies [114].

#### Antioxidants

In the search for improved pharmacological treatments for CRPS, the use of antioxidants has been increasingly studied over the past ten years, spanning the fields of rheumatology, anesthesiology and orthopedics. This is primarily regarding CRPS Type 1, with an established injury or trauma, in many cases from surgery. In fact, Eisenberg et al. in 2008 demonstrated that when comparing CRPS 1 and control patients, the patients with CRPS had significantly elevated salivary peroxidase, superoxide dismutase activity, uric acid and total antioxidant status compared to controls [115]. In 2014 Baykal et al. had similar findings, showing that superoxide dismutase, glutathione peroxidase and glutathione s-transferase activity were all significantly elevated in patients who developed CRPS I when compared to patients who did not [116]. Several studies have been done regarding the prophylactic treatment of surgical patients with Vitamin C post-operatively. These have found that prophylaxis with 500 mg to 1 g of Vitamin C daily post operatively from total knee arthroplasty, foot and ankle surgery, and subacromial shoulder surgery had a statistically significant effect on the development of CRPS 1 [117–119]. Patients who received Vitamin C were less likely to develop CRPS, considered to be through the role of Vitamin C in stabilizing reactive oxygen species and in turn reducing inflammation. Beyond this, a meta-analysis of randomized controlled trials using vitamin C for CRPS prophylaxis found a similar reduction in the risk of CRPS development [120]. In all, it is safe to say that Vitamin C perioperatively could be a valuable tool in the prevention of CRPS Type 1, especially in the elective surgical setting, ultimately reducing the incidence of this often-debilitating disease.

### Invasive interventions

#### Sympathetic blocks

A sympathetic block is a commonly used minimally invasive treatment for CRPS. Although frequently used, sympathetic blocks’ short- and long-term analgesic effects are not well supported by evidence. It is thought that one of the underlying pathophysiologic mechanisms of CRPS is sympathetic hyperactivity. The most common sympathetic blocks for CRPS are the stellate ganglion sympathetic blocks used to treat upper extremity symptoms while lumbar sympathetic nerve blocks are used to treat lower extremity symptoms [13]. Although the quality of the evidence was low, a 2013 Cochrane review found that sympathetic blocks combined with local anesthetic were ineffective at reducing CRPS-related pain [121]. A more recent Cochrane review in 2016 was unable to make any firm judgments regarding the effectiveness of this type of treatment for CRPS [121]. In 2019 a study looked at the relationship between sympathetic blocks and spinal cord stimulation and found that the effects of a sympathetic block did not predict the success of a spinal cord stimulator [122].

#### Spinal cord stimulation

With spinal cord stimulation (SCS), electrodes are positioned in the epidural space to deliver electric stimulation to the spinal cord’s dorsal column. Some devices use an external pulse generator, but typically the electrodes are connected to an implanted pulse generator. Several mechanisms of action for SCS have been proposed, including vasodilation, reversal of cortical maladaptive neuroplastic changes, adrenergic inhibition, and inhibition of nociceptive neural conduction in the spinal cord. A comprehensive outcome specific review of the use of spinal cord stimulation specifically for CRPS was published in 2016. High-level evidence (1B+) continues to support spinal cord stimulation’s beneficial and successful role in the treatment of CRPS patients’ perceived pain relief, pain score, and quality of life. Functional improvements were noted with less evidence for CRPS resolution, sleep hygiene improvement, positive psychological impact, and analgesic sparing effects [123]. A different study looking at the long-term outcome of SCS in CRPS did not find statistical functional improvement or a reduction in opioid or neuropathic pain medication use. However, they did note that 70% of the patients continued to use their SCS devices at the eight-year follow-up [124].

#### Dorsal root ganglion stimulation

An innovative neuromodulation technique for the treatment of chronic pain targets the dorsal root ganglion (DRG) rather than the spinal cord. The DRG serves as an ideal target given its role in processing and transmitting sensory information from the periphery to the central nervous system. In animal models of chronic pain, pathophysiologic changes in the DRG have been noted, including altered expression of various genes that may play a role in the hyperexcitability of neurons involved in the nociceptive pathway. The exact mechanism of how DRG stimulation exerts its beneficial effects remains unknown, although it may be related to the modulation of a variety of cells housed within the DRG [125–128].

As opposed to conventional SCS, DRG stimulation enables a more targeted application of neurostimulation due to the DRG’s more peripheral location and ease of access. For the treatment of lower extremity pain in CRPS, DRG stimulation received approval from the US Food and Drug Administration in 2016. According to a recent pooled analysis study, DRG stimulation was safe and effective for CRPS, resulting in a 4.9-point mean decrease in CRPS-I pain intensity [129]. In 2017 the ACCURATE study compared SCS and DRG stimulation in 152 patients with CRPS. DRG stimulation was found to be more efficient than conventional SCS in this multicenter randomized trial for reducing pain and enhancing quality of life in CRPS patients [127].

#### Amputation

The role of amputation in chronic CRPS type 1 is a potentially viable one, specifically for those patients who suffer from intractable and refractory symptoms despite numerous multi-modal therapeutic interventions. Although not without their own risks as well as the potential for recurrence of CRPS in the remaining limb or other limbs, several studies have noted patient satisfaction with the benefits of amputation. These retrospective reviews are relatively limited in power; however, they suggest a potential intervention for those patients who have exhausted alternative options [130–132]. There may be additional benefit in selecting potential amputation candidates for their resilience, as a study conducted by Bodde et al. found that patients with higher resilience scores correlated with higher quality of life and lower psychological distress status post limb amputation [90]. Regardless of the potential benefits, the decision to pursue amputation should be taken in the context of the patient overall, recognizing and acknowledging the associated complications of amputation as well as the risk for minimal relief to recurrence of CRPS.

#### Conclusion

CRPS is a debilitating, life-altering condition that remains ambiguous regarding its underlying pathophysiology although notable progress has been made. Treatment modalities are therefore directed at symptomatic management and targeting potential pathways with initial modalities comprised of non-invasive options such as PT, OT and psychological therapies in addition to neuropathic medications. For patients with persistent or refractory symptoms, more invasive and alternative therapies can be considered; however, consideration must be given to the inherent risks associated with more invasive techniques and the lack of robust, high-quality evidence to support their use. Therefore, although DRG has shown to be especially promising in recent studies, more research directed at both invasive and alternative therapies is necessary given the scarcity of clinical trials with significant patient populations being involved to ensure patient safety. As newer knowledge and advancements in the understanding of this condition expand, clinicians will be better equipped to target treatment modalities and manage these complex patients with an emphasis on early treatment initiation and adaptation of the rehabilitation program to ensure continued optimization for patients.

## Data Availability

Data sharing is not applicable to this article as no new data were created or analyzed in this study.

## References

[1] Goh EL, Chidambaram S, Ma D. Complex regional pain syndrome: a recent update. Burns Trauma. 2017;5(1):1. doi: 10.1186/S41038-016-0066-4. 28127572 PMC5244710

[2] Ott S, Maihöfner C. Signs and symptoms in 1,043 patients with complex regional pain syndrome. J Pain. 2018;19(6):599–17. doi: 10.1016/J.JPAIN.2018.01.004. 29409933

[3] Moretti A, Palomba A, Paoletta M, et al. Complex regional pain syndrome in athletes: scoping review. Medicina. 2021;57(11):1262. doi: 10.3390/medicina57111262. 34833480 PMC8623027

[4] Shim H, Rose J, Halle S, et al. Complex regional pain syndrome: a narrative review for the practising clinician. Br J Anaesth. 2019;123(2):e424–e433. doi: 10.1016/J.BJA.2019.03.03.0 31056241 PMC6676230

[5] Urits I, Shen AH, Jones MR, et al. Complex regional pain syndrome, current concepts and treatment options. Curr Pain Headache Rep. 2018;22(2):10. doi: 10.1007/S11916-018-0667-7/TABLES/2. 29404787

[6] Nazir SST, Ivan N, Antonella U, et al. Complex regional pain syndrome: a comprehensive review. Pain Ther. 2021;10(2):875–892. doi: 10.1007/S40122-021-00279-4. 34165690 PMC8586273

[7] Stanton-Hicks M, Jänig W, Hassenbusch S, et al. Reflex sympathetic dystrophy: changing concepts and taxonomy. Pain. 1995;63(1):127–133. doi: 10.1016/0304-3959(95)00110-E. 8577483

[8] Harden RN, Bruehl S, Stanton-Hicks M, et al. Proposed new diagnostic criteria for complex regional pain syndrome. Pain Med. 2007;8(4):326–331. doi: 10.1111/j.1526-4637.2006.00169.x. 17610454

[9] Knudsen LF, Terkelsen AJ, Drummond PD, et al. Complex regional pain syndrome: a focus on the autonomic nervous system. Clin Auton Res. 2019;29(4):457–467. doi: 10.1007/S10286-019-00612-0. 31104164

[10] David Clark J, Tawfik VL, Tajerian M, et al. Autoinflammatory and autoimmune contributions to complex regional pain syndrome. Mol Pain. 2018;14:1744806918799127. doi: 10.1177/1744806918799127. 30124090 PMC6125849

[11] Bruehl S, Maihöfner C, Stanton-Hicks M, et al. Complex regional pain syndrome: evidence for warm and cold subtypes in a large prospective clinical sample. Pain. 2016;157(8):1674–1681. doi: 10.1097/J.PAIN.0000000000000569. 27023422

[12] Ryskalin L, Ghelarducci G, Marinelli C, et al. Effectiveness of decision support to treat complex regional pain syndrome. Appl Sci. 2022;12(18):8979. doi: 10.3390/app12188979.

[13] Misidou C, Charalampos P, Mediterr J. Complex regional pain syndrome: an update. Mediterr J Rheumatol. 2019;30(1):16–25. doi: 10.31138/mjr.30.1.16. 32185338 PMC7045919

[14] Baronio M, Sadia H, Paolacci S, et al. Molecular aspects of regional pain syndrome. Pain Res Manag. 2020;2020:7697214. doi: 10.1155/2020/7697214. 32351641 PMC7171689

[15] Dirckx M, Stronks DL, Van Bodegraven-Hof EAM, et al. Inflammation in cold complex regional pain syndrome. Acta Anaesthesiol Scand. 2015;59(6):733–739. doi: 10.1111/AAS.12465. 25598133

[16] Sommer C, Leinders M, Üçeyler N. Inflammation in the pathophysiology of neuropathic pain. Pain. 2018;159(3):595–602. doi: 10.1097/J.PAIN.0000000000001122. 29447138

[17] Bickel M. The role of interleukin-8 in inflammation and mechanisms of regulation. J Periodontol. 1993;64(5 Suppl):456–460. 8315568

[18] Bernhard S, Hug S, Stratmann AEP, et al. Interleukin 8 elicits rapid physiological changes in neutrophils that are altered by inflammatory conditions. J Innate Immun. 2021;13(4):225–241. doi: 10.1159/000514885. 33857948 PMC8460987

[19] Bharwani KD, Dik WA, Dirckx M, et al. Highlighting the role of biomarkers of inflammation in the diagnosis and management of complex regional pain syndrome. Mol Diagn Ther. 2019;23(5):615–626. doi: 10.1007/S40291-019-00417-X/TABLES/4. 31363934 PMC6775035

[20] Russo MA, Georgius P, Pires AS, et al. Novel immune biomarkers in complex regional pain syndrome. J Neuroimmunol. 2020;347:577330. doi: 10.1016/j.jneuroim.2020. 32731051

[21] Alexander GM, Perreault MJ, Reichenberger ER, et al. Changes in immune and glial markers in the CSF of patients with complex regional pain syndrome. Brain Behav Immun. 2007;21(5):668–676. doi: 10.1016/J.BBI.2006.10.009. 17129705

[22] Alexander GM, Van Rijn MA, Van Hilten JJ, et al. Changes in cerebrospinal fluid levels of pro-inflammatory cytokines in CRPS. Pain. 2005;116(3):213–219. doi: 10.1016/J.PAIN.2005.04.013. 15964681

[23] Munts AG, Zijlstra FJ, Nibbering PH, et al. Analysis of cerebrospinal fluid inflammatory mediators in chronic complex regional pain syndrome related dystonia. Clin J Pain. 2008;24(1):30–34. doi: 10.1097/AJP.0B013E318156D961. 18180633

[24] Veldman PH, Reynen HM, Arntz IE, et al. Signs and symptoms of reflex sympathetic dystrophy: prospective study of 829 patients. Lancet. 1993;342(8878):1012–1016. doi: 10.1016/0140-6736(93)92877-v. 8105263

[25] Schinkel C, Gaertner A, Zaspel J, et al. Inflammatory mediators are altered in the acute phase of posttraumatic complex regional pain syndrome. Clin J Pain. 2006;22(3):235–239. doi: 10.1097/01.AJP.0000169669.70523.F0. 16514322

[26] Ribbers GM, Oosterhuis WP, Van Limbeek J, et al. Reflex sympathetic dystrophy: is the immune system involved? Arch Phys Med Rehabil. 1998;79(12):1549–1552. doi: 10.1016/S0003-9993(98)90418-X. 9862298

[27] Parkitny L, McAuley JH, Di Pietro F, et al. Inflammation in complex regional pain syndrome. Neurology. 2013;80(1):106–117 doi: 10.1212/WNL.0B013E31827B1AA1. 23267031 PMC3589200

[28] Wehmeyer C, Pap T, Buckley CD, et al. The role of stromal cells in inflammatory bone loss. Clin Exp Immunol. 2017;189(1):1–11. doi: 10.1111/CEI.12979. 28419440 PMC5461090

[29] Bertolini DR, Nedwin GE, Bringman TS, et al. Stimulation of bone resorption and inhibition of bone formation in vitro by human tumour necrosis factors. Nature. 1986;319(6053):516–518. doi: 10.1038/319516a0. 3511389

[30] Hashizume M, Hayakawa N, Mihara M. IL-6 trans-signalling directly induces RANKL on fibroblast-like synovial cells and is involved in RANKL induction by TNF-α and IL-17. Rheumatology. 2008;47(11):1635–1640. doi: 10.1093/RHEUMATOLOGY/KEN363. 18786965

[31] Littlejohn G. Neurogenic neuroinflammation in fibromyalgia and complex regional pain syndrome. Nat Rev Rheumatol. 2015;11(11):639–648. doi: 10.1038/nrrheum.2015.100. 26241184

[32] Birklein F, Drummond PD, Li W, et al. Activation of cutaneous immune responses in complex regional pain syndrome. J Pain. 2014;15(5):485–495. doi: 10.1016/j.jpain.2014.01.490. 24462502 PMC4011956

[33] Li WW, Guo TZ, Shi X, et al. Neuropeptide regulation of adaptive immunity in the tibia fracture model of complex regional pain syndrome. J Neuroinflammation. 2018;15(1):105. doi: 10.1186/S12974-018-1145-1/FIGURES/11. 29642930 PMC5896028

[34] Marinus J, Moseley GL, Birklein F, et al. Clinical features and pathophysiology of complex regional pain syndrome. Lancet Neurol. 2011;10(7):637–648. doi: 10.1016/S1474-4422(11)70106-5. 21683929 PMC5511749

[35] Kohr D, Tschernatsch M, Schmitz K, et al. Autoantibodies in complex regional pain syndrome bind to a differentiation-dependent neuronal surface autoantigen. Pain. 2009;143(3):246–251. doi: 10.1016/J.PAIN.2009.03.009. 19375222

[36] Tajerian M, Clark JD. New concepts in complex regional pain syndrome. Hand Clin. 2016;32(1):41–49. doi: 10.1016/J.HCL.2015.08.003. 26611388 PMC4662772

[37] Cuhadar U, Gentry C, Vastani N, et al. Autoantibodies produce pain in complex regional pain syndrome by sensitizing nociceptors. Pain. 2019;160(12):2855–2865. doi: 10.1097/J.PAIN.0000000000001662. 31343542

[38] Clatworthy MR, Aronin CEP, Mathews RJ, et al. Immune complexes stimulate CCR7-dependent dendritic cell migration to lymph nodes. Nat Med. 2014;20(12):1458–1463. doi: 10.1038/nm.3709. 25384086 PMC4283039

[39] Ratti C, Nordio A, Resmini G, et al. Post-traumatic complex regional pain syndrome: clinical features and epidemiology. Clin Cases Miner Bone Metab. 2015;12(Suppl 1):11–16. doi: 10.11138/CCMBM/2015.12.3S.011. PMC4832405 27134626

[40] Osborne S, Farrell J, Dearman RJ, et al. Cutaneous immunopathology of long-standing complex regional pain syndrome. Eur J Pain. 2015;19(10):1516–1526. doi: 10.1002/EJP.685. 25728589

[41] Russo M, Georgius P, Santarelli DM. A new hypothesis for the pathophysiology of complex regional pain syndrome. Med Hypotheses. 2018;119:41–53. doi: 10.1016/J.MEHY.2018.07.026. 30122490

[42] Villablanca EJ, Mora JR. A two-step model for langerhans cell migration to skin-draining LN. Eur J Immunol. 2008;38(11):2975–2980. doi: 10.1002/EJI.200838919. 18991275 PMC3204458

[43] Pleger B, Tegenthoff M, Ragert P, et al. Sensorimotor retuning [corrected] in complex regional pain syndrome parallels pain reduction. Ann Neurol. 2005;57(3):425–429. doi: 10.1002/ANA.20394. 15732114

[44] Sigtermans MJ, van Hilten JJ, Bauer MCR, et al. Ketamine produces effective and long-term pain relief in patients with complex regional pain syndrome type 1. Pain. 2009;145(3):304–311. doi: 10.1016/J.PAIN.2009.06.02.3 19604642

[45] Bruehl S. An update on the pathophysiology of complex regional pain syndrome. Anesthesiology. 2010;113(3):713–725. doi: 10.1097/ALN.0B013E3181E3DB38. 20693883

[46] Sommer C, Kress M. Recent findings on how proinflammatory cytokines cause pain: peripheral mechanisms in inflammatory and neuropathic hyperalgesia. Neurosci Lett. 2004;361(1-3):184–187. doi: 10.1016/j.neulet.2003.12.007. 15135924

[47] Lebel A, Becerra L, Wallin D, et al. fMRI reveals distinct CNS processing during symptomatic and recovered complex regional pain syndrome in children. Brain. 2008;131(Pt 7):1854–1879. doi: 10.1093/BRAIN/AWN123. 18567621

[48] Davis KD, Kiss ZHT, Tasker RR, et al. Thalamic stimulation-evoked sensations in chronic pain patients and in nonpain (movement disorder) patients. J Neurophysiol. 1996;75(3):1026–1037. doi: 10.1152/JN.1996.75.3.1026. 8867115

[49] Van Hilten JJ, Van de Beek WJT, Vein AA, et al. Clinical aspects of multifocal or generalized tonic dystonia in reflex sympathetic dystrophy. Neurology. 2001;56(12):1762–1765. doi: 10.1212/WNL.56.12.1762. 11425951

[50] Le Bars D, Cadden SW. What is a wide-dynamic-range cell? The Senses: a Comprehensive Reference. 2008;5:331–338. doi: 10.1016/B978-012370880-9.00167-5.

[51] Vera-Portocarrero LP, Zhang ET, Ossipov MH, et al. Descending facilitation from the rostral ventromedial medulla maintains nerve injury-induced central sensitization. Neuroscience. 2006;140(4):1311–1320. doi: 10.1016/J.NEUROSCIENCE.2006.03.016. 16650614

[52] Harden RN, Rudin NJ, Bruehl S, et al. Increased systemic catecholamines in complex regional pain syndrome and relationship to psychological factors: a pilot study. Anesth Analg. 2004;99(5):1478–1485. doi: 10.1213/01.ANE.0000132549.25154.ED. 15502052

[53] Bruehl S, Chung OY. Psychological and behavioral aspects of complex regional pain syndrome management. Clin J Pain. 2006;22(5):430–437. doi: 10.1097/01.AJP.0000194282.82002.79. 16772797

[54] Reimer M, Rempe T, Diedrichs C, et al. Sensitization of the nociceptive system in complex regional pain syndrome. PLOS One. 2016;11(5):e0154553. doi: 10.1371/JOURNAL.PONE.0154553. 27149519 PMC4858201

[55] Drummond PD. Neuronal changes resulting in up-regulation of alpha-1 adrenoceptors after peripheral nerve injury. Neural Regen Res. 2014;9(14):1337–1340. doi: 10.4103/1673-5374.137583. 25221588 PMC4160862

[56] Johnson T. The collected works of ambroise paré. New York: Milford House; 1968.

[57] Denmark A. An example of symptoms resembling tic douleureux, produced by a wound in the radial nerve. Med Chir Trans. 1813;4:48–52. PMC2128932 20895207

[58] Mitchell SW, Morehouse GR, Keen WW. Gunshot wounds and other injuries of nerves. Clin Orthop Relat Res. 2007;458:35–39. doi: 10.1097/BLO.0b013e31803df02c. 17473596

[59] Stanton-Hicks M. CRPS: what’s in a name? Taxonomy, epidemiology, neurologic, immune and autoimmune considerations. Reg Anesth Pain Med. 2019;44(3):376–387. doi: 10.1136/rapm-2018-100064. 30777902

[60] Leriche R. De la causalgie envisagee comme une nervrite du sympathique et son traitement par la denuation et l’excision des plexus nerveux periarteriels. Presse Med. 1916;24:178–180.

[61] Kessler A, Yoo M, Calisoff R, et al. Complex regional pain syndrome: an updated comprehensive review. NeuroRehabilitation. 2020;47(3):253–264. doi: 10.3233/NRE-208001. 32986618

[62] Evans JA. Reflex sympathetic dystrophy. Surg Clin North Am. 1946;26:780–790. 20988032

[63] Castillo-Guzmán S, Nava-Obregón TA, Palacios-Ríos D, et al. Complex regional pain syndrome (CRPS), a review. Medicina Universitaria. 2015;17(67):114–121. doi: 10.1016/j.rmu.2015.03.003.

[64] Classification of chronic pain. Descriptions of chronic pain syndromes and definitions of pain terms. Prepared by the international association for the study of pain, subcommittee on taxonomy. Pain Suppl. 1986;3:S1–S226. 3461421

[65] Williams K, Guarino A, Raja SN. Chapter 27 – Complex regional pain syndrome. In: Benzon HT, Raja SN, Liu SS, et al., eds. Essentials of pain medicine. 4th ed. Philadelphia: Elsevier; 2018:223.e2–232.e2. doi: 10.1016/B978-0-323-40196-8.00027-9.

[66] Harden RN, Bruehl S, Perez RSGM, et al. Validation of proposed diagnostic criteria (the “budapest criteria”) for complex regional pain syndrome. Pain. 2010;150(2):268–274. doi: 10.1016/j.pain.2010.04.030. 20493633 PMC2914601

[67] Goebel A, Birklein F, Brunner F, et al. The Valencia consensus-based adaptation of the IASP complex regional pain syndrome diagnostic criteria. Pain. 2021;162(9):2346–2348. doi: 10.1097/j.pain.0000000000002245. 33729210 PMC8374712

[68] Mesaroli G, Hundert A, Birnie KA, et al. Screening and diagnostic tools for complex regional pain syndrome: a systematic review. Pain. 2021;162(5):1295–1304. doi: 10.1097/j.pain.0000000000002146. 33230004 PMC8054537

[69] Tahmoush AJ. Causalgia: redefinition as a clinical pain syndrome. Pain. 1981;10(2):187–197. doi: 10.1016/0304-3959(81)90194-9. 7267135

[70] Sandroni P, Low PA, Ferrer T, et al. Complex regional pain syndrome I (CRPS I): prospective study and laboratory evaluation. Clin J Pain. 1998;14(4):282–289. doi: 10.1097/00002508-199812000-00003. 9874005

[71] Thomson McBride AR, Barnett AJ, Livingstone JA, et al. Complex regional pain syndrome (type 1): a comparison of 2 diagnostic criteria methods. Clin J Pain. 2008;24(7):637–640. doi: 10.1097/AJP.0b013e31817591da. 18716503

[72] Sumitani M, Shibata M, Sakaue G, et al. Development of comprehensive diagnostic criteria for complex regional pain syndrome in the Japanese population. Pain. 2010;150(2):243–249. doi: 10.1016/j.pain.2010.03.03.2 20451323

[73] Dommerholt J. Complex regional pain syndrome—1: history, diagnostic criteria and etiology. J Bodyw Mov Ther. 2004;8(3):167–177. doi: 10.1016/S1360-8592(03)00100-1.

[74] Goebel A, Barker C, Birklein F, et al. Standards for the diagnosis and management of complex regional pain syndrome: results of a European pain federation task force. Eur J Pain. 2019;23(4):641–651. doi: 10.1002/ejp.1362. 30620109 PMC6593444

[75] Shin SH, Kim SJ. Bone scintigraphy in patients with pain. Korean J Pain. 2017;30(3):165–175. doi: 10.3344/kjp.2017.30.3.165. 28757916 PMC5532523

[76] Ringer R, Wertli M, Bachmann LM, et al. Concordance of qualitative bone scintigraphy results with presence of clinical complex regional pain syndrome 1: meta‐analysis of test accuracy studies. Eur J Pain. 2012;16(10):1347–1356. doi: 10.1002/j.1532-2149.2012.00137.x. 22473897

[77] Wertli MM, Brunner F, Steurer J, et al. Usefulness of bone scintigraphy for the diagnosis of complex regional pain syndrome 1: a systematic review and Bayesian meta-analysis. PLOS One. 2017;12(3):e0173688. doi: 10.1371/journal.pone.0173688. 28301606 PMC5354289

[78] Pérez-Concha T, Tijero B, Acera M, et al. Usefulness of thermography in the diagnosis and classification of complex regional pain syndrome. Neurología. 2020;38(5):342–349. doi: 10.1016/j.nrl.2020.10.011. 37263729

[79] Lubkowska A, Pluta W. Infrared thermography as a Non-Invasive tool in musculoskeletal disease rehabilitation–The control variables in applicability–A systematic review. Appl Sci. 2022;12(9):4302. doi: 10.3390/app12094302.

[80] Bruehl S, Harden RN, Galer BS, et al. Complex regional pain syndrome: are there distinct subtypes and sequential stages of the syndrome? Pain. 2002;95(1-2):119–124. doi: 10.1016/s0304-3959(01)00387-6. 11790474

[81] Collins CK. Physical therapy management of complex regional pain syndrome I in a 14-year-old patient using strain counterstrain: a case report. J Man Manip Ther. 2007;15(1):25–41 doi: 10.1179/106698107791090150. 19066641 PMC2565594

[82] Menck JY, Requejo SM, Kulig K. Thoracic spine dysfunction in upper extremity complex regional pain syndrome type I. J Orthop Sports Phys Ther. 2000;30(7):401–409. doi: 10.2519/JOSPT.2000.30.7.401. 10907896

[83] Cleland J, McRae M. Complex regional pain syndrome I: management through the use of vertebral and sympathetic trunk mobilization. J Man Manip Ther. 2002;10(4):188–199. doi: 10.1179/106698102790819067.

[84] Gutiérrez-Espinoza H, Tabach-Apraiz A, Oyanadel-Maldonado M. Physical therapy in patients with complex regional pain syndrome type I after distal radius fracture: a case series. J Phys Ther Sci. 2019;31(4):403–407. doi: 10.1589/JPTS.31.40.3 31037018 PMC6451956

[85] Walston Z, Hernandez L, Yake D. Utilization of manual therapy to the lumbar spine in conjunction with traditional conservative care for individuals with bilateral lower extremity complex regional pain syndrome: A case series. Physiother Theory Pract. 2018;36(1):241–248. doi: 10.1080/09593985.2018.1482392. 29873592

[86] Albazaz R, Wong YT, Homer-Vanniasinkam S. Complex regional pain syndrome: a review. Ann Vasc Surg. 2008;22(2):297–306. doi: 10.1016/J.AVSG.2007.10.006. 18346583

[87] Fialka V, Korpan M, Saradeth T, et al. Autogenic training for reflex sympathetic dystrophy: a pilot study. Complement Ther Med. 1996;4(2):103–105. doi: 10.1016/S0965-2299(96)80026-4.

[88] Lohnberg JA, Altmaier EM. A review of psychosocial factors in complex regional pain syndrome. J Clin Psychol Med Settings. 2013;20(2):247–254. doi: 10.1007/S10880-012-9322-3. 22961122

[89] Edwards RR, Smith MT, Kudel I, et al. Pain-related catastrophizing as a risk factor for suicidal ideation in chronic pain. Pain. 2006;126(1-3):272–279. doi: 10.1016/J.PAIN.2006.07.004. 16926068

[90] Bodde MI, Schrier E, Krans HK, et al. Resilience in patients with amputation because of complex regional pain syndrome type I. Disabil Rehabil. 2014;36(10):838–843. doi: 10.3109/09638288.2013.822023. 23930642

[91] Tang NKY, Crane C. Suicidality in chronic pain: a review of the prevalence, risk factors and psychological links. Psychol Med. 2006;36(5):575–586. doi: 10.1017/S0033291705006859. 16420727

[92] Mackey S, Feinberg S. Pharmacologic therapies for complex regional pain syndrome. Curr Pain Headache Rep. 2007;11(1):38–43. doi: 10.1007/S11916-007-0020-Z. 17214920 PMC2914598

[93] Resmini G, Ratti C, Canton G, et al. Treatment of complex regional pain syndrome. Clin Cases Miner Bone Metab. 2015;12(Suppl 1):26–30. doi: 10.11138/CCMBM/2015.12.3S.026. 27134629 PMC4832403

[94] Kalita J, Vajpayee A, Misra UK. Comparison of prednisolone with piroxicam in complex regional pain syndrome following stroke: a randomized controlled trial. QJM. 2006;99(2):89–95. doi: 10.1093/QJMED/HCL004. 16428335

[95] Atalay N, Ercidogan O, Akkaya N, et al. Prednisolone in complex regional pain syndrome. Pain Phys. 2014;2;17(2;3):179–185. doi: 10.36076/ppj.2014/17/179. 24658479

[96] Bianchi C, Rossi S, Turi S, et al. Long-term functional outcome measures in corticosteroid-treted complex regional pain syndrome. Eur J Phys Rehabil Med. 2006;42(2):103–111. 16767061

[97] Jamroz A, Berger M, Winston P. Prednisone for acute complex regional pain syndrome: a retrospective cohort study. Pain Res Manag. 2020;2020:8182569. doi: 10.1155/2020/8182569. 32184912 PMC7060858

[98] Gelman B, Hsu ES. Gabapentin. In: Sinatra RS, Jahr JS, J. Watkins-Pitchford M (editors). The essence of analgesia and analgesics. Cambridge: Cambridge University Press; 2022. p. 294–297. doi: 10.1017/CBO9780511841378.071.

[99] van de Vusse AC, Stomp-van den Berg SGM, Kessels AHF, et al. Randomised controlled trial of gabapentin in complex regional pain syndrome type 1 [ISRCTN84121379]. BMC Neurol. 2004;4(1):13. doi: 10.1186/1471-2377-4-13. 15453912 PMC523854

[100] Brown S, Johnston B, Amaria K, et al. A randomized controlled trial of amitriptyline versus gabapentin for complex regional pain syndrome type I and neuropathic pain in children. Scand J Pain. 2016;13(1):156–163. doi: 10.1016/J.SJPAIN.2016.05.039. 28850523

[101] Varenna M, Adami S, Sinigaglia L. Bisphosphonates in complex regional pain syndrome type I: how do they work? Clin Exp Rheumatol. 2014;32(4):451–454. 24959990

[102] Chevreau M, Romand X, Gaudin P, et al. Bisphosphonates for treatment of complex regional pain syndrome type 1: a systematic literature review and meta-analysis of randomized controlled trials versus placebo. Joint Bone Spine. 2017;84(4):393–399. doi: 10.1016/J.JBSPIN.2017.03.009. 28408275

[103] Varenna M, Adami S, Rossini M, et al. Treatment of complex regional pain syndrome type I with neridronate: a randomized, double-blind, placebo-controlled study. Rheumatology. 2013;52(3):534–542. doi: 10.1093/rheumatology/kes3.12 23204550

[104] Rosenbaum SB, Gupta V, Patel P, et al. Ketamine. In: StatPearls [Internet]. Treasure Island (FL): StatPearls Publishing; 2024.

[105] Connolly SB, Prager JP, Harden RN. A systematic review of ketamine for complex regional pain syndrome. Pain Med. 2015;16(5):943–969. doi: 10.1111/PME.12675. 25586192

[106] Azari P, Lindsay DR, Briones D, et al. Efficacy and safety of ketamine in patients with complex regional pain syndrome. CNS Drugs. 2012;26(3):215–228. doi: 10.2165/11595200-000000000-00000. 22136149

[107] Wissel J, Müller J, Dressnandt J, et al. Management of spasticity associated pain with botulinum toxin A. J Pain Symptom Manage. 2000;20(1):44–49. doi: 10.1016/S0885-3924(00)00146-9. 10946168

[108] Schilder JCM, Van Dijk JG, Dressler D, et al. Responsiveness to botulinum toxin type a in muscles of complex regional pain patients with tonic dystonia. J Neural Transm. 2014;121(7):761–767. doi: 10.1007/S00702-014-1172-8/FIGURES/2. 24532257

[109] Carroll I, Clark JD, Mackey S. Sympathetic block with botulinum toxin to treat complex regional pain syndrome. Ann Neurol. 2009;65(3):348–351. doi: 10.1002/ANA.21601. 19334078 PMC2763598

[110] Siongco PRL, Rosales RL, Moore AP, et al. Botulinum neurotoxin injections for muscle-based (dystonia and spasticity) and non-muscle-based (neuropathic pain) pain disorders: a meta-analytic study. J Neural Transm. 2020;127(6):935–951. doi: 10.1007/S00702-020-02163-5/TABLES/7. 32146504

[111] Lee Y, Lee CJ, Choi E, et al. Lumbar sympathetic block with botulinum toxin type a and type B for the complex regional pain syndrome. Toxins. 2018;10(4):164. doi: 10.3390/TOXINS10040164. 29671801 PMC5923330

[112] Comella CL, Jankovic J, Shannon KM, et al. Comparison of botulinum toxin serotypes a and B for the treatment of cervical dystonia. Neurology. 2005;65(9):1423–1429. doi: 10.1212/01.WNL.0000183055.81056.5C. 16275831

[113] Tokuno H. Efficacy of Botulinum toxin injection in reducing limb pain in patients with complex regional pain syndrome. https://clinicaltrials.gov/ct2/show/NCT03616262

[114] Singh V, Cohen SP. Prolonging sympathetic blockade for complex regional pain syndrome: is botulinum toxin the answer? Anesthesiology. 2022;136(2):261–264. doi: 10.1097/ALN.0000000000004093. 34965301 PMC9054445

[115] Eisenberg E, Shtahl S, Geller R, et al. Serum and salivary oxidative analysis in complex regional pain syndrome. Pain. 2008;138(1):226–232. doi: 10.1016/J.PAIN.2008.04.019. 18539395

[116] Baykal T, Seferoglu B, Karsan O, et al. Antioxidant profile in patients with complex regional pain syndrome type I. Int J Rheum Dis. 2014;17(2):156–158. doi: 10.1111/1756-185X.12140. 24576270

[117] Hernigou J, Labadens A, Ghistelinck B, et al. Vitamin C prevention of complex regional pain syndrome after foot and ankle surgery: a prospective randomized study of three hundred and twenty nine patients. Int Orthop. 2021;45(9):2453–2459. doi: 10.1007/S00264-021-05159-2/TABLES/3. 34347132

[118] Jacques H, Jérôme V, Antoine C, et al. Prospective randomized study of the vitamin C effect on pain and complex pain regional syndrome after total knee arthroplasty. Int Orthop. 2021;45(5):1155–1162. doi: 10.1007/S00264-020-04936-9/TABLES/4. 33438072

[119] Laumonerie P, Martel M, Tibbo ME, et al. Influence of vitamin C on the incidence of CRPS-I after subacromial shoulder surgery. Eur J Orthop Surg Traumatol. 2020;30(2):221–226. doi: 10.1007/S00590-019-02542-Z/TABLES/3. 31541301

[120] Aïm F, Klouche S, Frison A, et al. Efficacy of vitamin C in preventing complex regional pain syndrome after wrist fracture: a systematic review and meta-analysis. Orthop Traumatol Surg Res. 2017;103(3):465–470. doi: 10.1016/J.OTSR.2016.12.021. 28274883

[121] O’Connell NE, Wand BM, Gibson W, et al. Local anaesthetic sympathetic blockade for complex regional pain syndrome. Cochrane Database Syst Rev. 2016;7(7):CD004598. doi: 10.1002/14651858.CD004598. 27467116 PMC7202132

[122] Cheng J, Salmasi V, You J, et al. Outcomes of sympathetic blocks in the management of complex regional pain syndrome: a retrospective cohort study. Anesthesiology. 2019;131(4):883–893. doi: 10.1097/ALN.0000000000002899. 31365367

[123] Visnjevac O, Costandi S, Patel BA, et al. A comprehensive Outcome-Specific review of the use of spinal cord stimulation for complex regional pain syndrome. Pain Pract. 2017;17(4):533–545. doi: 10.1111/PAPR.12513. 27739179

[124] Hoikkanen T, Nissen M, Ikäheimo TM, et al. Long-Term outcome of spinal cord stimulation in complex regional pain syndrome. Neurosurgery. 2021;89(4):597–609. doi: 10.1093/NEUROS/NYA.B239 34245150 PMC8440061

[125] Graham RD, Sankarasubramanian V, Lempka SF. Dorsal root ganglion stimulation for chronic pain: hypothesized mechanisms of action. J Pain. 2022;23(2):196–211. doi: 10.1016/J.JPAIN.2021.07.008. 34425252 PMC8943693

[126] Koetsier E, Franken G, Debets J, et al. Mechanism of dorsal root ganglion stimulation for pain relief in painful diabetic polyneuropathy is not dependent on GABA release in the dorsal horn of the spinal cord. CNS Neurosci Ther. 2020;26(1):136–143. doi: 10.1111/CNS.13192. 31334605 PMC6930820

[127] Deer TR, Levy RM, Kramer J, et al. Dorsal root ganglion stimulation yielded higher treatment success rate for complex regional pain syndrome and causalgia at 3 and 12 months: a randomized comparative trial. Pain. 2017;158(4):669–681. doi: 10.1097/J.PAIN.0000000000000814. 28030470 PMC5359787

[128] Koopmeiners AS, Mueller S, Kramer J, et al. Effect of electrical field stimulation on dorsal root ganglion neuronal function. Neuromodulation. 2013;16(4):304–311. doi: 10.1111/NER.12028. 23421796

[129] Huygen FJPM, Liem L, Nijhuis H, et al. Evaluating dorsal root ganglion stimulation in a prospective Dutch cohort. Neuromodulation. 2019;22(1):80–86. doi: 10.1111/NER.12798. 30079622

[130] Krans-Schreuder HK, Bodde MI, Schrier E, et al. Amputation for long-standing, therapy-resistant type-I complex regional pain syndrome. J Bone Joint Surg Am. 2012;94(24):2263–2268. doi: 10.2106/JBJS.L.00532. 23318617

[131] Honkamp N, Amendola A, Hurwitz S, et al. Retrospective review of eighteen patients who underwent transtibial amputation for intractable pain. J Bone Joint Surg Am. 2001;83(10):1479–1483. doi: 10.2106/00004623-200110000-00003. 11679596

[132] Kashy BK, Abd-Elsayed AA, Farag E, et al. Amputation as an unusual treatment for Therapy-Resistant complex regional pain syndrome, type 1. Ochsner J. 2015;15(4):441–442. doi: 10.1043/TOJ-14-0074. 26730230 PMC4679307

